# MiR‐503 suppresses fibroblast activation and myofibroblast differentiation by targeting VEGFA and FGFR1 in silica‐induced pulmonary fibrosis

**DOI:** 10.1111/jcmm.16051

**Published:** 2020-11-01

**Authors:** Qiuyun Wu, Lei Han, Wenwen Gui, Feng Wang, Weiwen Yan, Hua Jiang

**Affiliations:** ^1^ School of Public Health Xuzhou Medical University Xuzhou China; ^2^ Institute of Occupational Disease Prevention Jiangsu Provincial Center for Disease Control and Prevention Nanjing China; ^3^ Tianjin Institute of Environmental and Operational Medicine Tianjin China; ^4^ Department of Occupational Medicine and Environmental Health Key Laboratory of Modern Toxicology of Ministry of Education School of Public Health Nanjing Medical University Nanjing China

**Keywords:** miR‐503, myofibroblast differentiation, pulmonary fibrosis, silicosis

## Abstract

Inhalation and deposition of crystalline silica particles in the lung can cause pulmonary fibrosis, then leading to silicosis. Given the paucity of effective drugs for silicosis, new insights for understanding the mechanisms of silicosis, including lung fibroblast activation and myofibroblast differentiation, are essential to explore therapeutic strategies. Our previous research showed that the up‐regulation of miR‐503 alleviated silica‐induced pulmonary fibrosis in mice. In this study, we investigated whether miR‐503 can regulate the TGF‐β1‐induced effects in lung fibroblasts. Mimic‐based strategies aiming at up‐regulating miR‐503 were used to discuss the function of miR‐503 in vivo and in vitro. We found that the expression level of miR‐503 was decreased in fibroblasts stimulated by TGF‐β1, and the up‐regulation of miR‐503 reduced the release of fibrotic factors and inhibited the migration and invasion abilities of fibroblasts. Combined with the up‐regulation of miR‐503 in a mouse model of silica‐induced pulmonary fibrosis, we revealed that miR‐503 mitigated the TGF‐β1‐induced effects in fibroblasts by regulating VEGFA and FGFR1 and then affecting the MAPK/ERK signalling pathway. In conclusion, miR‐503 exerted protective roles in silica‐induced pulmonary fibrosis and may represent a novel and potent candidate for therapeutic strategies in silicosis.

## 1 INTRODUCTION

Silicosis is a progressive fibrotic lung disease caused by inhalation of silica particles for a long time.^1^ Silica particles can activate alveolar macrophages and damage epithelial cells to release a large number of pro‐fibrotic factors (TGF‐β1, CTGF, PDGF, etc).^2^, ^3^ Such factors facilitate the activation and proliferation of lung fibroblasts, which further secrete extracellular matrix, leading to pulmonary fibrosis.^4^, ^5^, ^6^ Therefore, targeting lung fibroblasts may be a feasible strategy for developing new antifibrotic drugs for silicosis.

MiRNAs, a kind of small non‐coding RNA with ~22 nucleotides in length, have attracted much attention because of their wide participation in a variety of biological processes by regulating target genes.^7^ The abnormal expression of miRNAs and the disorder of intracellular signal cascade are closely correlated with the pathological process of pulmonary fibrosis.^8^ For example, the expression level of miR‐4508 from peripheral blood lymphocytes was lower in subjects with silica‐related pulmonary fibrosis than that in healthy controls.^9^ Elevated expression of miR‐7 relieved silica‐induced pulmonary fibrosis by blocking epithelial‐mesenchymal transition progress.^10^ Our previous study also indicated that miR‐1224‐5p targeted BECN1 to regulate mitochondrial damage in silica‐induced pulmonary fibrosis.^11^ Besides, overexpression of miR‐489 inhibited Smad3 and its mediated TGF‐β signal to alleviate silica‐induced pulmonary fibrosis.^12^ These preliminary studies indicated that miRNAs may be new targets for the development of effective drugs for silicosis.

MiR‐503 is located in Xq26.3 and belongs to the miR‐16 family.^13^ Our previous research showed that the up‐regulation of miR‐503 alleviated silica‐induced pulmonary fibrosis in mice.^14^ Therefore, it is necessary to further explore the regulatory mechanism of miR‐503 in the pathological process of silicosis. In this study, we found that miR‐503 negatively modulated the TGF‐β1‐induced effects in lung fibroblasts. Then, a series of tests in vivo and in vitro was designed to illustrate how miR‐503 regulates the TGF‐β1‐induced effects in fibroblasts and to examine the antifibrogenic potential of miR‐503 in silica‐induced pulmonary fibrosis. Eventually, we demonstrated that miR‐503 may represent a new therapeutic strategy for the treatment of silicosis.

## 2 MATERIALS AND METHODS

### 2.1 Cell treatment and transfection

The human lung fibroblast cells (MRC‐5) were kindly provided by Stem Cell Bank, Chinese Academy of Sciences (Shanghai, China), and cultured in minimum essential medium (MEM, Life Technologies/Gibco, Grand Island, NY, USA) containing 10% foetal bovine serum (FBS, Life Technologies/Gibco, Grand Island, NY, USA), 100 U/mL penicillin and 100 μg/mL streptomycin (Beyotime Bio, Shanghai, China). MRC‐5 cells have typical fibroblastic morphology and present the characteristics of fibroblasts in the pathological process of pulmonary fibrosis.^15^, ^16^ Therefore, this cell line is widely used in the study of the fibrogenesis process.^8^, ^17^ For TGF‐β1 stimulation, MRC‐5 cells (3 × 10^5^ cells/well) were seeded into 6‐well plates overnight and then incubated with TGF‐β1 (Sigma‐Aldrich, St. Louis, MO, USA) for 48 hours. The total RNA and protein were extracted for further experiments.

For transfection analysis, the miR‐503 mimic and mimic control (miR‐NC), the siRNAs for VEGFA, FGFR1 and negative control siRNA (si‐NC) were designed and synthesized by RiboBio Co., Ltd. (Guangzhou, China). The fibroblast cells (3 × 10^5^ cells/well) were cultured in 6‐well plates for 24 hours and then transfected with miRNA mimic or siRNA using riboFECT^™^ CP Reagent (RiboBio Co., Ltd., Guangzhou, China) according to the manufacturer's protocol. The VEGFA and FGFR1 plasmids were transfected using Lipofectamine 2000 reagent (Invitrogen, Carlsbad, USA). The cells were treated with TGF‐β1 at 24 hours after transfection and harvested for further experiments after 48 hours.

### 2.2 Immunofluorescence

The fibroblasts were seeded in a 4‐chamber glass‐bottom dish (Cellvis) and then treated with TGF‐β1 for 48 hours. After fixing with carbinol^18^ and blocking with 5% BSA, the fibroblasts were incubated with the primary antibodies (ɑ‐SMA, Abcam, ab32575, 1:500) at 4°C overnight and with Cy3‐conjugated goat anti‐rabbit secondary antibodies (1:200, Beyotime Bio, Shanghai, China) at room temperature for 1 hour. The nucleus was labelled with DAPI (Beyotime Bio, Shanghai, China) for 10 minutes. The fibroblasts were imaged with a fluorescence microscope (Olympus, Tokyo, Japan).

### 2.3 Western blot assay

Western blot assay was performed as previously described.^11^ The expression levels of collagen I (Abcam, ab138492, 1:2000), ɑ‐SMA (Abcam, ab32575, 1:2000), vimentin (Cell Signaling Technology, 5741, 1:1000), VEGFA (Abcam, ab52917, 1:2000), FGFR1 (Cell Signaling Technology, 9740, 1:1000), total ERK 1/2 (Cell Signaling Technology, 9102, 1:1000), phospho‐ERK 1/2 (Cell Signaling Technology, 9101, 1:1000) and GAPDH (Beyotime Bio, Shanghai, China) were measured by Western blot assay. The density of protein expression was relatively quantified by the ImageJ software.

### 2.4 Quantitative real‐time PCR assay

RNA isolation and qRT‐PCR assay were described previously.^11^ The Bulge Loop^™^ miRNA qRT‐PCR Primer Set (one RT primer and a pair of qPCR primers for each set) specific for miR‐503 was designed by RiboBio Co., Ltd. (Guangzhou, China). The relative expression levels of miR‐503 were normalized to the levels of U6.

### 2.5 Luciferase assay

The firefly luciferase reporter plasmid containing VEGFA and FGFR1 3’UTR‐WT and 3’UTR‐Mut were created from the psiCHECK‐2 vector (Generay Biotech Co., Ltd., Shanghai, China). According to the manufacturer's protocol, a total of 400 ng of each plasmid together with 25 ng Renilla luciferase construct (pRL‐SV40) were transfected into fibroblasts; then, 30 nmol/L miR‐503 or mimic control (miR‐NC) was transfected using reagent (RiboBio Co., Ltd., Guangzhou, China) and cultured for 24 hours. The Renilla and firefly luciferase activities were determined using a dual‐luciferase assay system according to the manufacturer's instructions.

### 2.6 Scratch assay

The migration ability of fibroblasts was evaluated using the scratch assay. The cells (5 × 10^5^ cells/well) were seeded in 6‐well plates. Until 70%‐80% coverage, the cross‐shaped scratch across the cell monolayer was made by a sterile pipette tip gently. After washing to remove the debris with PBS, the cells were cultured in serum‐free medium. The digital images of scratches were captured at 0 and 24 hours. The area of the cell gap was determined by the ImageJ software. The following equation was used to evaluate the migrated area (%): [(cell gap at 0 hour − cell gap after 24 hours)/cell gap at 0 hour] × 100%.

### 2.7 Invasion assay

The invasion ability of fibroblasts was observed by using 24‐well BioCoat Matrigel Invasion Chamber (Corning, NY, USA). After transfecting with miRNA mimic, the cells (1 × 10^5^ cells/well) were added to the upper chamber. The bottom wells of the chamber were filled with normal medium, whereas the fibroblasts in the upper chamber were treated with TGF‐β1. After 48 hours, the non‐invading cells on the top of the membrane were removed with a cotton swab. The invading cells on the back of the membrane were fixed with methanol for 20 minutes and dyed with 0.1% crystal violet solution for 15 minutes. Then, the images were obtained by optical microscopy.

### 2.8 Cell viability assay

The fibroblasts were seeded in 96‐well plates at approximately 1,000 cells/well. Then, the cells were transfected with miR‐503 mimic for 24 hours and incubated with TGF‐β1 for another 48 hours. Then, 10% CCK‐8 reagent (Beyotime Bio, Shanghai, China) diluted in the normal medium was added to 96‐well plates. After incubating for 4 hours at 37°C, the OD value was measured using an automatic microplate reader (TECAN Infinite M200, Männedorf, Switzerland).

### 2.9 Animal experiments

C57BL/6 male mice (4‐6 weeks of age) were purchased from SLAC Laboratory Animal Co., Ltd., (Shanghai, China). All experimental protocols were approved by the Animal Care and Use Committee at Nanjing Medical University. The mice were anaesthetized with an intraperitoneal injection of 1% pentobarbital sodium (Dainippon Sumitomo Pharma, Osaka, Japan). A 0.05 mL sterile saline containing silica (50 mg/kg, Sigma‐Aldrich, St. Louis, MO, USA) was directly instilled intratracheally. The control group was instilled with 0.05 mL saline. The lung tissues were harvested on day 7, 14 or 28 after silica instillation. The miR‐503 up‐regulation mouse model was conducted by a co‐instillation of 200 nmol/kg miR‐503 agomir (RiboBio Co., Ltd., Guangzhou, China) with silica instillation. Subsequently, 120 nmol/kg miR‐503 agomir was injected via the tail vein weekly. The lung tissues were harvested at day 28.

### 2.10 Statistical analysis

All data were expressed as the means ± SD of at least three independent experiments. Data were analysed using independent‐samples *t* tests between two groups and one‐way analysis of variance (ANOVA) for more groups with Dunnett's test. A value of *P* < .05 was considered significant.

## 3 RESULTS

### 3.1 TGF‐β1 decreased miR‐503 expression in lung fibroblasts

**Figure 1:**
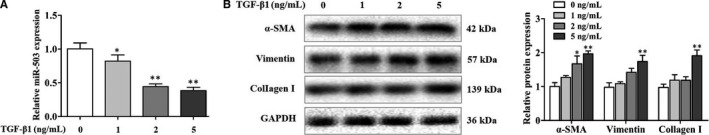
TGF‐β1 decreased miR‐503 expression in lung fibroblasts. (A) qRT‐PCR assay of miR‐503 levels in fibroblasts treated with different doses of TGF‐β1 for 48 h, with *P < .05 and **P < .01 vs the dose 0 group. (B) Western blot assay of the protein expression levels of α‐SMA, vimentin and collagen I in fibroblasts treated with different doses of TGF‐β1 for 48 h, with *P < .05 and **P < .01 vs the dose 0 group

Our previous study showed that the early up‐regulation of miR‐503 alleviated silica‐induced pulmonary fibrosis in mice.^14^ Fibroblast effects, such as activation and myofibroblast differentiation, are the key pathological stages of silica‐induced pulmonary fibrosis. TGF‐β1 released by macrophages is a potent mediator in the myofibroblast differentiation process. However, the effects of miR‐503 on TGF‐β1‐stimulated fibroblast responses in silicosis have rarely been reported. To discuss this issue, we used different doses of TGF‐β1 to stimulate lung fibroblasts. As shown in Figure 1A, the levels of miR‐503 were significantly decreased in fibroblasts with the rising dosage of TGF‐β1, reaching the lowest level at 5 ng/mL. So we chose 5 ng/mL TGF‐β1 to treat cells in further experiments. Next, we found that the protein expression levels of ɑ‐SMA, vimentin and collagen I were increased after TGF‐β1 stimulation (Figure 1B). These changes are regarded as typical signs of fibroblast responses in silicosis. Thus, we suggested that miR‐503 may play an important role in fibroblast effects of silica‐induced pulmonary fibrosis.

### 3.2 Enhanced expression of miR‐503 attenuated the TGF‐β1‐induced effects in fibroblasts

**Figure 2:**
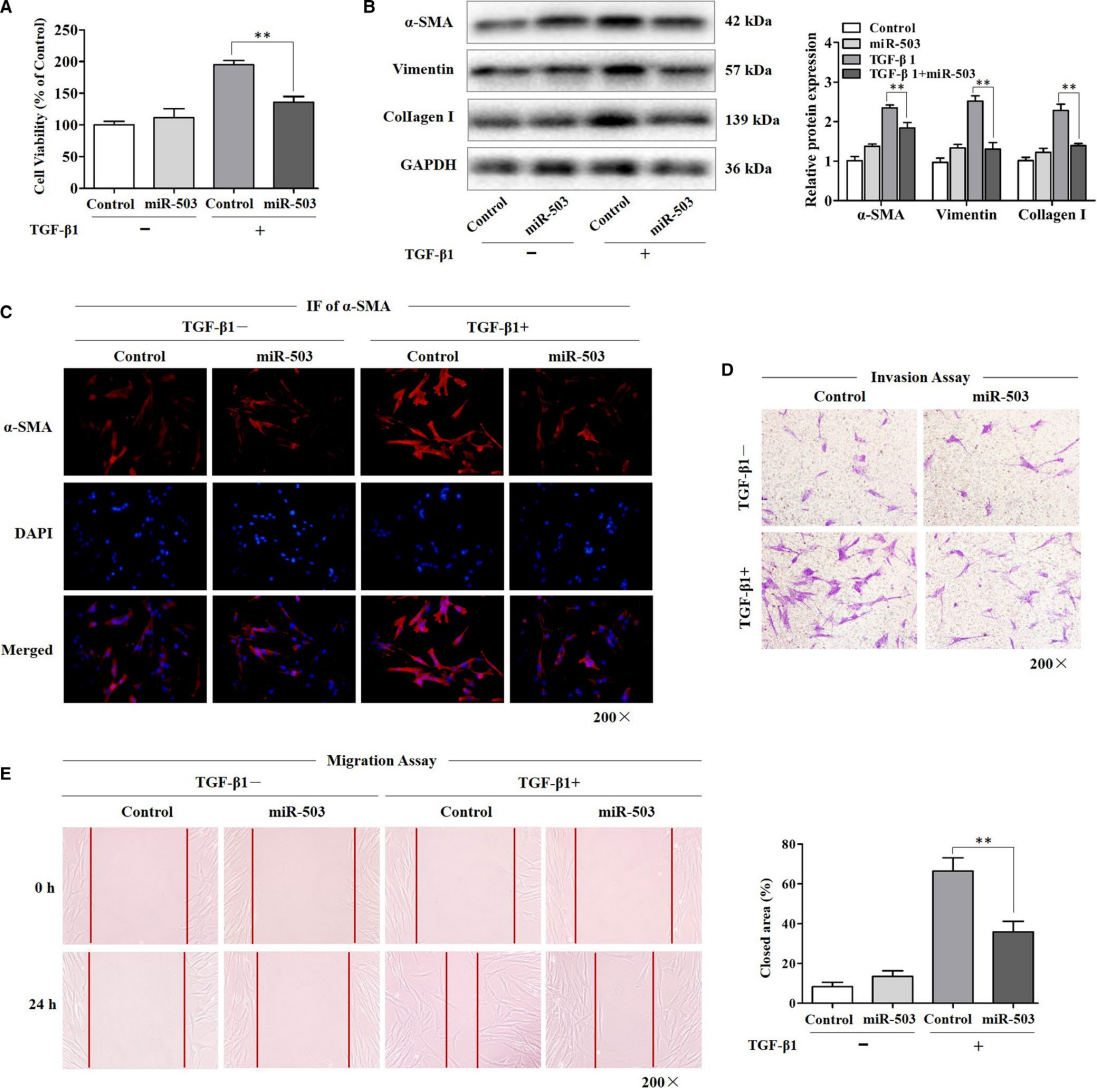
Enhanced expression of miR‐503 attenuated the TGF‐β1‐induced effects in fibroblasts. (A) The viability of fibroblasts was observed by CCK‐8 assay. The fibroblasts were transfected with miR‐503 mimic and then treated with 5 ng/mL TGF‐β1 for 48 h, with **P < .01 vs the TGF‐β1 group. (B) Western blot assay of the protein expression levels of α‐SMA, vimentin and collagen I in fibroblasts transfected with miR‐503 mimic, with **P < .01 vs the TGF‐β1 group. (C) The photographs of fluorescence staining in fibroblasts transfected with miR‐503 mimic. TGF‐β1 treatment was started 24 h after transfection, and staining was performed after 48‐h treatment. The expression of α‐SMA was detected using an anti‐α‐SMA antibody, and DAPI was used for nuclear staining. Magnification, 200×. (D) The invasion ability of fibroblasts transfected with miR‐503 mimic was detected using the invasion chamber experiment. Magnification, 200×. (E) The migration ability of fibroblasts was detected using the scratch experiment. The closed area (%) was calculated by equation: [(cell gap at 0 h ‐ cell gap after 24 h)/cell gap at 0 h] ×100%, with **P < .01 vs the TGF‐β1 group. Magnification, 200×

To further investigate the functional roles of miR‐503 in regulating TGF‐β1‐induced effects in fibroblasts, we evaluated the proliferation of fibroblasts with the up‐regulation of miR‐503. As shown in Figure 2A, CCK‐8 assay displayed that transfection of fibroblasts with miR‐503 mimic had an obvious inhibitory effect on TGF‐β1‐induced fibroblast proliferation. Moreover, the enhanced expression of miR‐503 reduced the protein expression levels of ɑ‐SMA, vimentin and collagen I (Figure 2B) and weakened the immunofluorescence activity of α‐SMA induced by TGF‐β1 (Figure 2C). Besides, we found that the increased invasion and migration abilities of fibroblasts induced by TGF‐β1 were also suppressed by the overexpression of miR‐503 (Figure 2D,E). All of these results pointed out that TGF‐β1 triggered fibroblast effects, whereas miR‐503 inhibited these effects, indicating the antifibrotic properties of miR‐503.

### 3.3 MiR‐503 regulated VEGFA and FGFR1 in vivo and in vitro

**Figure 3:**
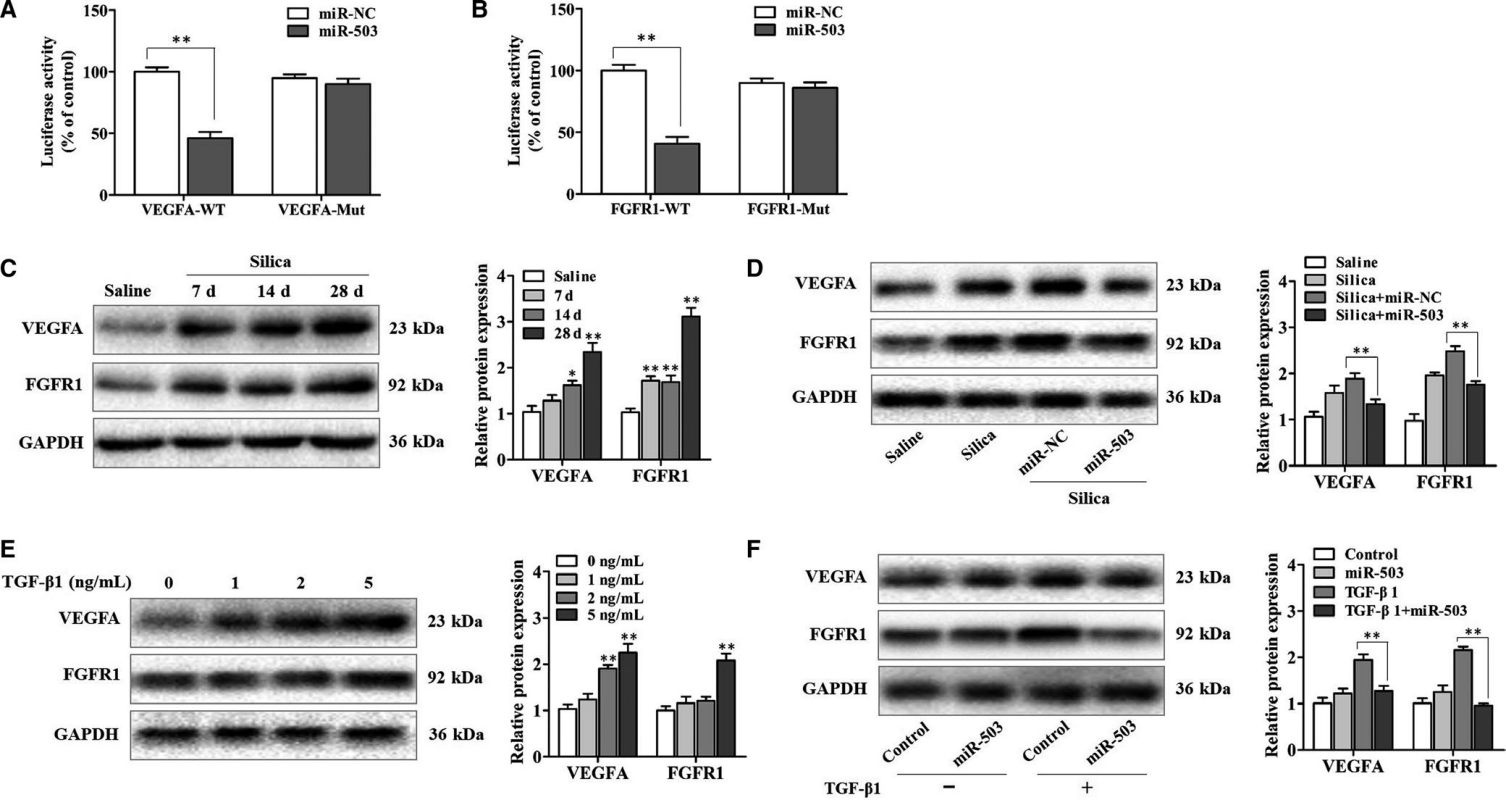
MiR‐503 regulated VEGFA and FGFR1 in vivo and in vitro. (A) The relative luciferase activity of fibroblasts transfected with VEGFA‐WT and VEGFA‐Mut plasmids, with **P < .01 vs the miR‐NC group. (B) The relative luciferase activity of fibroblasts transfected with FGFR1‐WT and FGFR1‐Mut plasmids, with **P < .01 vs the miR‐NC group. (C) Western blot assay of VEGFA and FGFR1 expression in mouse lung tissues on days 7, 14 and 28 after a single intratracheal instillation of silica particles, with *P < .05 and **P < .01 vs the saline group. (D) Western blot assay of VEGFA and FGFR1 expression in the lung tissues of miR‐503 up‐regulated mouse model, with **P < .01 vs the silica plus miR‐NC group. (E) The protein expression levels of VEGFA and FGFR1 in fibroblasts treated with different doses of TGF‐β1 for 48 h, with **P < .01 vs the dose 0 group. (F) The protein expression levels of VEGFA and FGFR1 in fibroblasts transfected with miR‐503 mimic and then treated with TGF‐β1 for 48 h, with **P < .01 vs the TGF‐β1 group

After confirming the roles of miR‐503 in the TGF‐β1‐mediated fibroblast effects, we next identified the target gene to clarify the underlying mechanisms responsible for the antifibrotic roles of miR‐503. In this study, we found that VEGFA and FGFR1 may be potential miR‐503 targets. Given these two targets were functionally related to several pathways in the fibrogenesis process, we hypothesized that VEGFA and FGFR1 likely act in concert to promote TGF‐β1‐induced fibroblast effects and that miR‐503 fine‐tunes this regulatory signal. Therefore, we performed a luciferase reporter assay to reveal the bind of miR‐503 to VEGFA and FGFR1, respectively. As illustrated in Figure 3A,B, miR‐503 restrained the luciferase activity of wild‐type VEGFA and FGFR1 reporter in fibroblasts. However, miR‐503 lost its inhibitory effect with mutated VEGFA and FGFR1, which established that both VEGFA and FGFR1 were miR‐503 targets. Besides, we found that the protein expression levels of VEGFA and FGFR1 were significantly up‐regulated in the lung tissues of silica‐treated mice (Figure 3C), and overexpression of miR‐503 in a mouse model of silicosis efficiently suppressed these two targets (Figure 3D). Furthermore, the protein expression levels of VEGFA and FGFR1 were increased as the concentration of TGF‐β1 rose in fibroblasts (Figure 3E), and miR‐503 overexpression also reduced the levels of these two targets (Figure 3F). These data indicated that VEGFA and FGFR1 were involved in fibroblast responses in silicosis, and miR‐503 regulated VEGFA and FGFR1 in vivo and in vitro.

### 3.4 The partnership of VEGFA and FGFR1 in the TGF‐β1‐induced effects in fibroblasts

**Figure 4:**
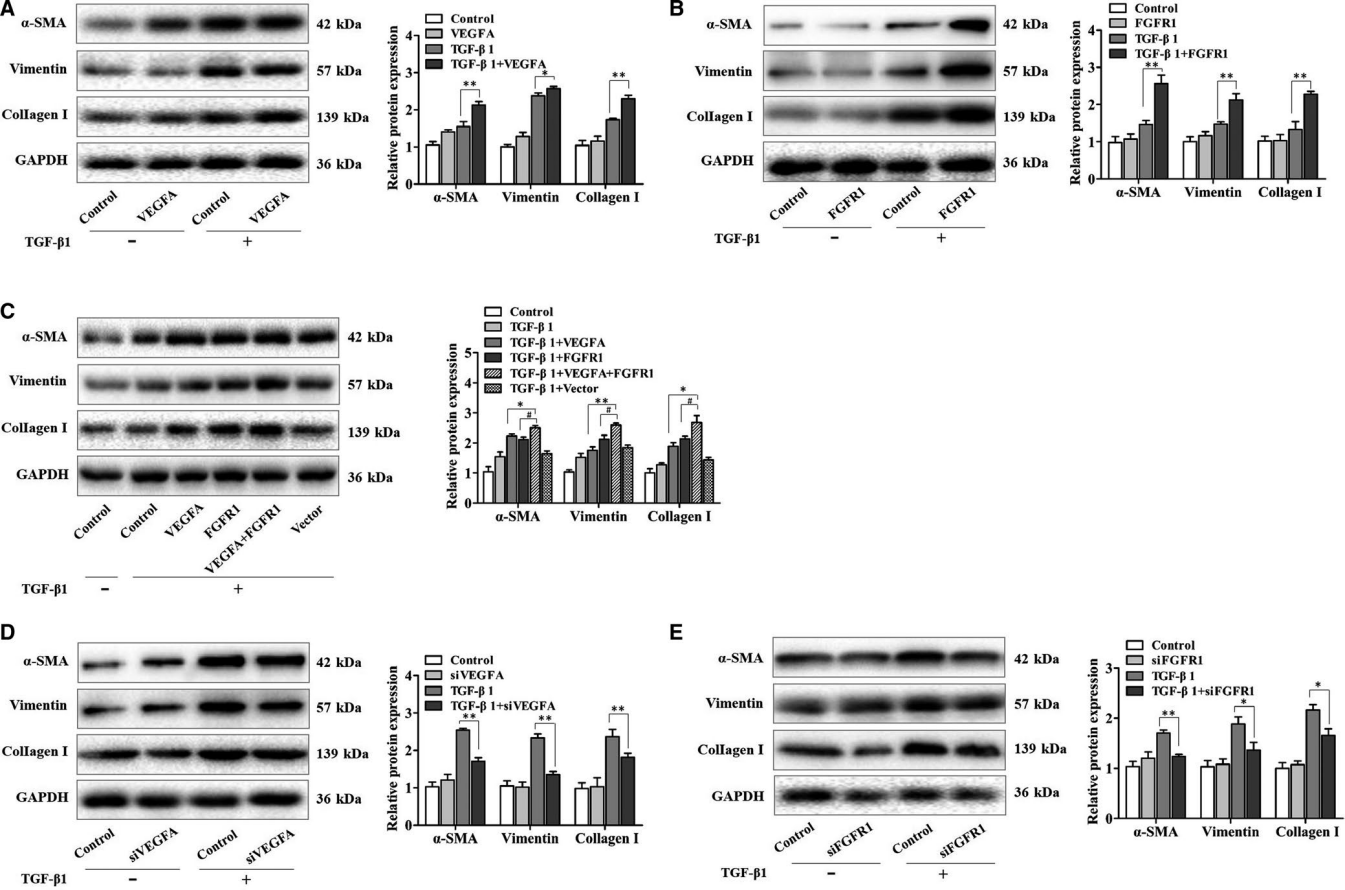
The partnership of VEGFA and FGFR1 in the TGF‐β1‐induced effects in fibroblasts. (A, B) The protein expression levels of α‐SMA, vimentin and collagen I in fibroblasts transfected with VEGFA or FGFR1 plasmid, respectively, with *P < .05 and **P < .01 vs the TGF‐β1 group. (C) The protein expression levels of α‐SMA, vimentin and collagen I in fibroblasts transfected with VEGFA and FGFR1 plasmids, with *P < .05 and **P < .01 vs the TGF‐β1 plus VEGFA group and #*P* < .05 vs the TGF‐β1 plus FGFR1 group. (D, E) The protein expression levels of α‐SMA, vimentin and collagen I in fibroblasts transfected with VEGFA or FGFR1 siRNA, respectively, with *P < .05 and **P < .01 vs the TGF‐β1 group

To discuss the biological functions of VEGFA and FGFR1 in TGF‐β1‐treated fibroblast, plasmid and siRNA transfection assays were performed. We found that the activated protein expression levels of ɑ‐SMA, vimentin and collagen I in fibroblasts were further enhanced by overexpression of VEGFA or FGFR1, respectively (Figure 4A,B). Additionally, we observed that the signs of enhanced effects were much more obvious in fibroblasts transfected with the combination of VEGFA and FGFR1 plasmids compared with the transfection of VEGFA or FGFR1 plasmid alone (Figure 4C). These results indicated that the synergistic effects of VEGFA and FGFR1 were responsible for more severe fibroblast reactions. Meanwhile, a siRNA against VEGFA or FGFR1 significantly prevented the expression levels of ɑ‐SMA, vimentin and collagen I in TGF‐β1‐treated fibroblasts (Figure 4D,E). Taken together, we proposed that VEGFA and FGFR1 served as a co‐operator in the TGF‐β1‐induced effects in fibroblast.

### 3.5 MiR‐503 regulated ERK1/2 activation in TGF‐β1‐treated fibroblasts by targeting VEGFA and FGFR1

**Figure 5:**
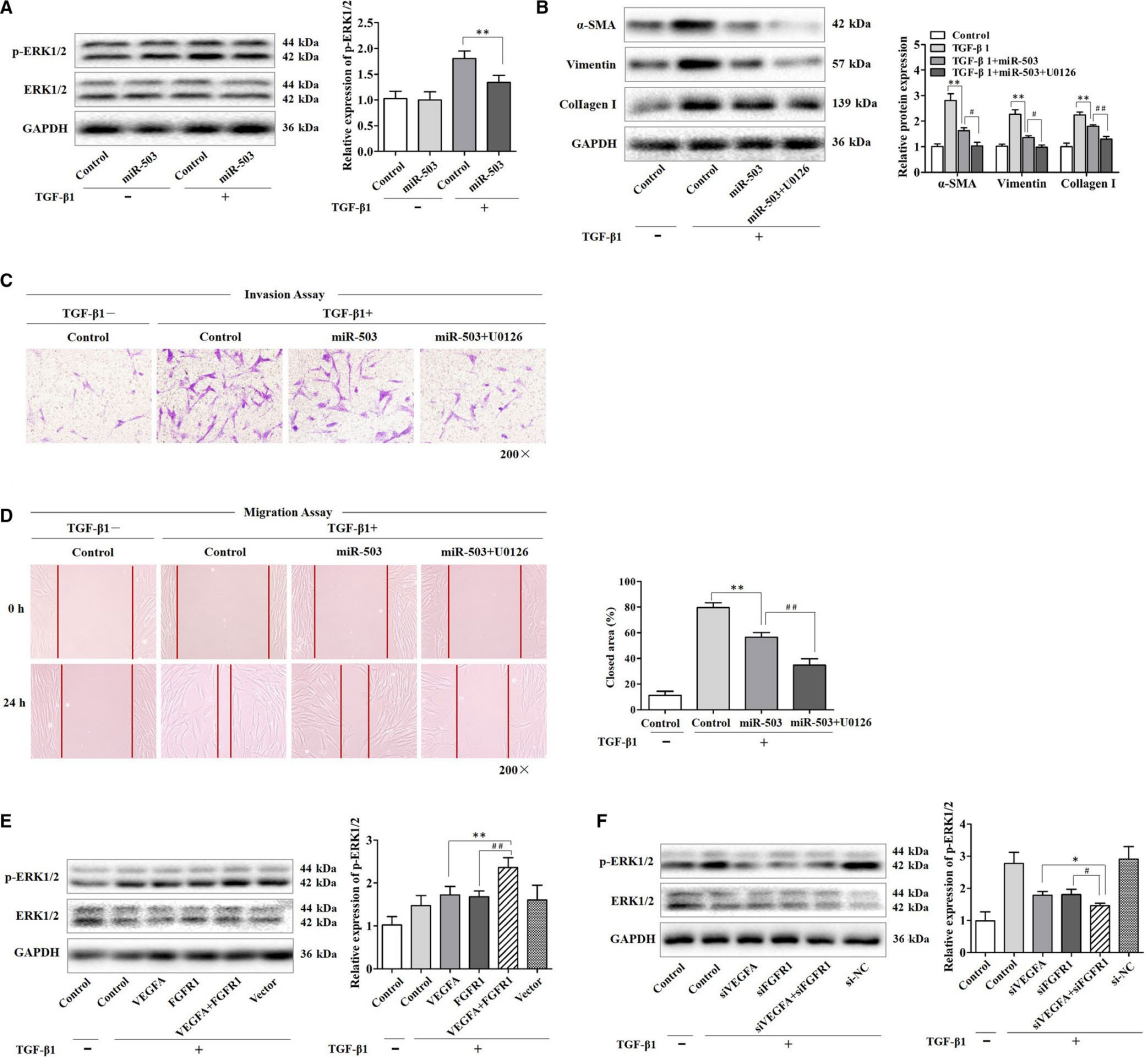
MiR‐503 regulated ERK1/2 activation in TGF‐β1‐treated fibroblasts by targeting VEGFA and FGFR1. (A) The protein expression levels of phospho‐ERK1/2 (p‐ERK1/2) and ERK1/2 in fibroblasts transfected with miR‐503 mimic, with **P < .01 vs the TGF‐β1 group. (B) The protein expression levels of α‐SMA, vimentin and collagen I in fibroblasts transfected with miR‐503 mimic. A MEK inhibitor U0126 (20 μmol/L) treatment was started at 12 h after transfection and then treated with TGF‐β1 for 48 h, with **P < .01 vs the TGF‐β1 group and with #*P* < .05 and ##*P* < .01 vs the TGF‐β1 plus miR‐503 group. (C, D) The invasion and migration abilities of fibroblasts were detected. The closed area (%) was calculated by equation: [(cell gap at 0 h − cell gap after 24 h)/cell gap at 0 h] × 100%, with **P < .01 vs the TGF‐β1 group and ##*P* < .01 vs the TGF‐β1 plus miR‐503 group. Magnification, 200×. (E) The protein expression levels of p‐ERK1/2 and ERK1/2 in fibroblasts transfected with VEGFA and FGFR1 plasmids and then treated with TGF‐β1 for 48 h, with **P < .01 vs the TGF‐β1 plus VEGFA group and ##*P* < .01 vs the TGF‐β1 plus FGFR1 group. (F) The protein expression levels of p‐ERK1/2 and ERK1/2 in fibroblasts transfected with VEGFA and FGFR1 siRNAs, with *P < .05 vs the TGF‐β1 plus siVEGFA group and #*P* < .05 vs the TGF‐β1 plus siFGFR1 group

The initiation and maintenance of fibroblast fibrogenic response are currently viewed as the result of a complex network, with the crosstalk of the MAPK/ERK pathway playing a significant role. Previous studies supported that VEGFA and FGFR1 were involved in MAPK/ERK signalling.^19^, ^20^ However, the roles of MAPK/ERK signalling in fibroblasts are not well understood. Therefore, we then investigated whether miR‐503 affects ERK1/2 phosphorylation in fibroblasts treated with TGF‐β1. It showed that the fibroblasts exposed to TGF‐β1 exhibited elevated ERK1/2 phosphorylation, likely secondary to the reduction of miR‐503 expression. This can be explained by the reason that the up‐regulation of miR‐503 in these cells led to a significant inhibition in TGF‐β1‐induced ERK1/2 phosphorylation (Figure 5A). Furthermore, U0126 (20 μmol/L), a MEK inhibitor, was used to test the biological effect of miR‐503 on the MAPK/ERK signalling pathway. We found that pre‐treatment fibroblasts with U0126 exerted a more obvious inhibitory effects of ɑ‐SMA, vimentin and collagen I in the presence of miR‐503 mimic transfection (Figure 5B), which also further retarded the invasion and migration abilities of fibroblasts repressed by miR‐503 overexpression (Figure 5C,D). These results provided evidence supporting the role of the MAPK/ERK pathway in the TGF‐β1‐induced effects in fibroblasts. Additionally, overexpression of VEGFA and FGFR1 promoted the ERK1/2 phosphorylation in TGF‐β1‐treated fibroblasts, whereas knockdown of VEGFA and FGFR1 further inhibited these effects (Figure 5E,F). Finally, we demonstrated a prominent role of miR‐503 in the regulation of TGF‐β1‐induced effects in fibroblasts by targeting VEGFA and FGFR1, and then the MAPK/ERK pathway.

## 4 DISCUSSION

MiRNAs play significant roles in the pathogenic process of fibrogenesis and may represent valuable targets for the treatment of silicosis.^21^, ^22^, ^23^ Here, we found that miR‐503 was a negative modulator in the process of the TGF‐β1‐induced fibroblast actions. Furthermore, these antifibrotic effects of miR‐503 were because of the regulation of VEGFA and FGFR1, and then the inhibition of MAPK/ERK signalling pathway.

Researches showed that miR‐503 regulated EIF4E to prevent the proliferation of hepatocellular carcinoma cells,^24^ and the low expression of miR‐503 was closely related to the poor prognosis of patients with gastric cancer.^25^ However, different opinions indicated that reducing miR‐503 levels alleviated myocardial fibrosis by up‐regulating Apelin‐13.^26^ We also measured the levels of Apelin‐13, and it showed no significant changes (Figure S1). Therefore, miR‐503 may play different roles in various diseases. In this study, we observed that miR‐503 was significantly decreased in TGF‐β1‐stimulated fibroblasts, and the up‐regulation of miR‐503 mitigated fibroblasts effects. On this basis, further exploration of the molecular mechanism of miR‐503 will provide a new target for the treatment of silicosis.

VEGFA, known as vascular endothelial growth factor A, is a member of the VEGF family.^27^, ^28^ We found that VEGFA was a target gene of miR‐503, which was consistent with other studies.^29^, ^30^ It has been reported that inhibition of VEGF prevented angiogenesis and vascular leakage, thus slowing down bleomycin‐induced pulmonary fibrosis.^31^, ^32^ Additionally, cigarette extract induced VEGFA release in lung fibroblasts.^33^ Our results showed that the levels of VEGFA were increased both in the lung tissues of silicotic mice and in TGF‐β1‐treated fibroblasts, and miR‐503 negatively regulated VEGFA expression. Furthermore, VEGF promoted the expression of TGF‐β1 and α‐SMA.^34^ We found that VEGFA facilitated the secretion of fibrotic factors in TGF‐β1‐treated fibroblasts. Therefore, the down‐regulation of miR‐503 resulted in the release of target VEGFA and the amplification of downstream signals.

VEGF can interact with FGFR1 in the process of liver fibrosis.^35^ FGFR1 is a member of the FGFR family and a key receptor of the FGF pathway.^36^, ^37^, ^38^ We found that FGFR1 was another target gene of miR‐503, which was also supported in the published literature.^39^ The role of FGF/FGFRs signals in pulmonary fibrosis is still debatable. On the one hand, FGF1/FGFR1 and downstream PI3K and MAPK signals were enhanced in the lung tissues of patients with end‐stage IPF, and the expression of FGFR1 was increased in primary lung fibroblasts treated with TGF‐β1.^40^ On the other hand, FGF/FGFRs signals promoted epithelial cell survival and inhibited fibroblast differentiation to protect IPF.^41^ Despite different viewpoints, the FGFR1‐mediated signal was closely related to pulmonary fibrosis. We found that FGFR1 was also increased both in the lung tissues of silicotic mice and in TGF‐β1‐treated fibroblasts and negatively regulated by miR‐503. Meanwhile, FGFR1 promoted the secretion of fibrotic factors in TGF‐β1‐treated fibroblasts. Therefore, in addition to serving as an independent target gene, whether VEGFA and FGFR1 exert an associated pro‐fibrotic function is worthy of further study.

FGF/FGFRs signals and VEGF pathway play a synergistic role in promoting angiogenesis. For example, VEGF promoted FGF2 expression in endothelial cells, while blocking FGFR1 reduced VEGF levels.^42^ MiRNAs are known to regulate certain diseases by co‐targeting multiple genes or co‐operating with multiple miRNAs.^43^, ^44^ However, it is not clear whether miR‐503 plays a regulatory role by jointly targeting VEGFA and FGFR1 in silica‐induced pulmonary fibrosis and whether there is a synergistic effect between VEGFA and FGFR1. In this study, we found that miR‐503 functionally co‐targeted VEGFA and FGFR1 in the TGF‐β1‐induced effects in fibroblast. Overexpression of both VEGFA and FGFR1 further increased fibrotic molecule levels. Moreover, the combination of VEGFA and FGFR1 activated the important MAPK/ERK signalling pathway, whose pro‐fibrotic role has been widely described.^45^, ^46^ Therefore, we reasoned that miR‐503 negatively regulated the TGF‐β1‐induced effects in fibroblast by co‐targeting VEGFA and FGFR1 to affect the MAPK/ERK pathway, thus alleviating silica‐induced pulmonary fibrosis. Our findings represented a better understanding of the co‐regulation of multiple genes by the same miRNA in silicosis. Further researches in vivo will be necessary to clarify the precise regulation of VEGFA and FGFR1 by miR‐503 in silicosis.

**Figure 6:**
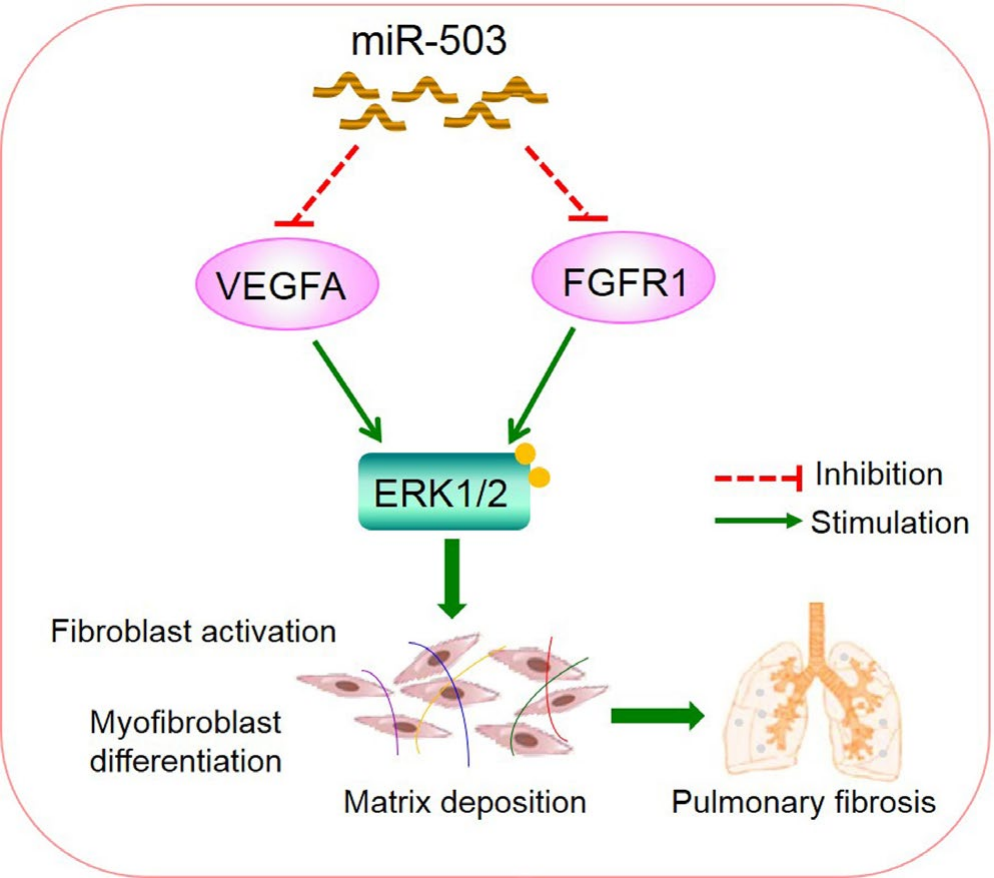
Schematic diagram showing the mechanisms by which miR‐503 regulated TGF‐β1‐induced effects in fibroblasts. MiR‐503 co‐targeted VEGFA and FGFR1, which led to block MAPK/ERK signalling and postpone fibroblast activation and myofibroblast differentiation reaction, thereby attenuated silica‐induced pulmonary fibrosis

In conclusion, we found that miR‐503 was a negative modulator of the TGF‐β1‐induced effects in fibroblasts. Mechanistically, miR‐503 co‐targeted VEGFA and FGFR1, and then blocked the MAPK/ERK pathway, thus alleviating silica‐induced pulmonary fibrosis (Figure 6). Our findings provided direct evidence that miR‐503 may represent a promising target for antifibrotic drug development of silicosis.

## CONFLICT OF INTEREST

The authors confirm that there are no conflicts of interest.

## AUTHOR CONTRIBUTIONS

**Qiuyun Wu:** Conceptualization (equal); Project administration (equal); Writing‐original draft (equal); Writing‐review & editing (equal). **Lei Han:** Conceptualization (equal); Writing‐review & editing (equal). **Wenwen Gui:** Project administration (equal); Writing‐review & editing (equal). **Feng Wang:** Data curation (equal); Writing‐original draft (equal); Writing‐review & editing (equal). **Weiwen Yan:** Methodology (equal); Writing‐review & editing (equal). **Hua Jiang:** Data curation (equal); Writing‐review & editing (equal).

## Supporting information

Fig S1

## Data Availability

The data are available from the corresponding author on a reasonable request.
