# Supplementary figures and images for: MiR‐503 suppresses fibroblast activation and myofibroblast differentiation by targeting VEGFA and FGFR1 in silica‐induced pulmonary fibrosis
Source: J Cell Mol Med. 2020 Nov 1;24(24):14339–48. doi: 10.1111/jcmm.16051 (PMC7754009; doi:10.1111/jcmm.16051)

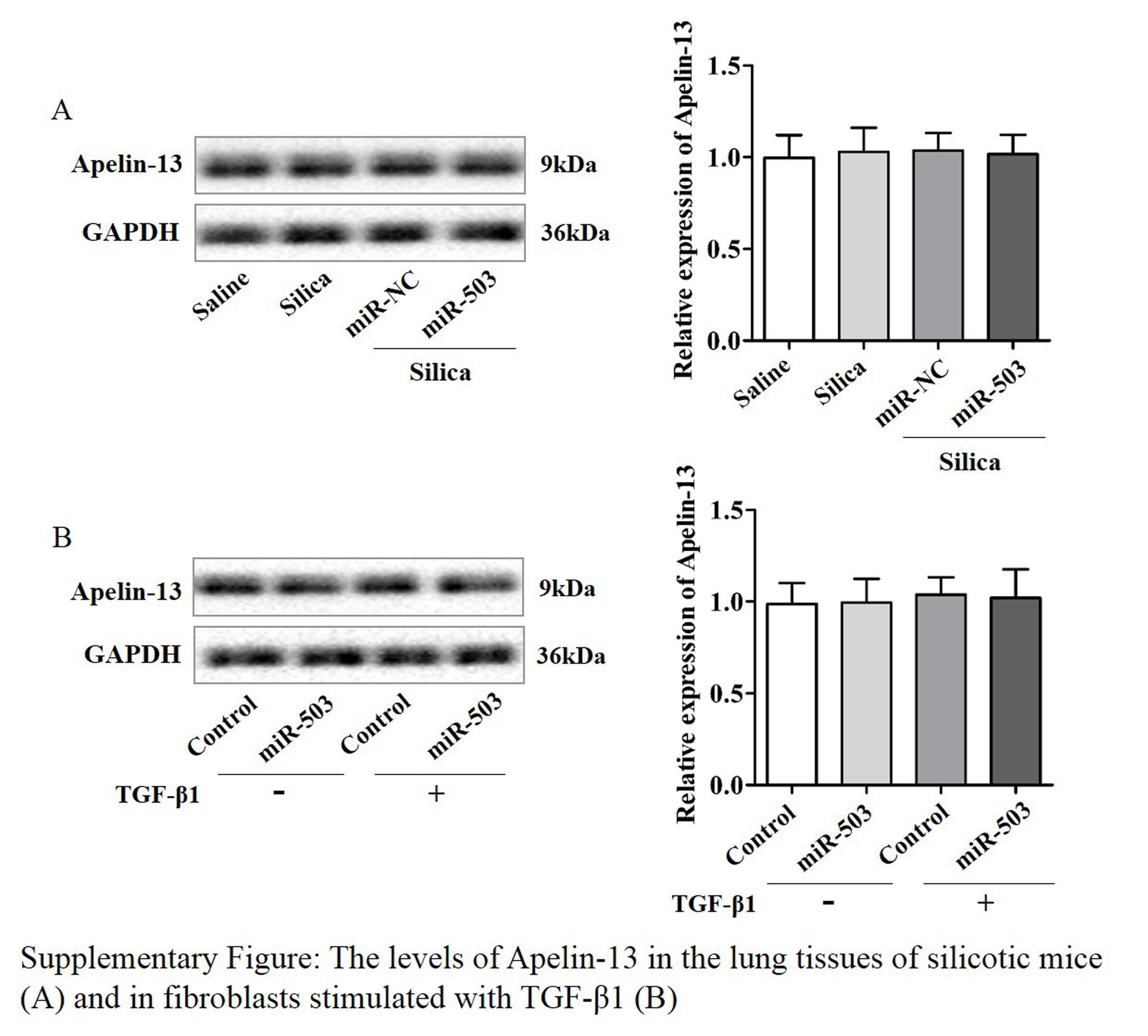

Supplement: Supplementary file 1 — Fig S1 [file JCMM-24-14339-s001.jpg]
